# Changes in the incidence and prevalence of human immunodeficiency virus or acquired immunodeficiency syndrome in the South African medical schemes environment: 2005–2015

**DOI:** 10.4102/sajhivmed.v21i1.1007

**Published:** 2020-06-29

**Authors:** Floidy Wafawanaka, Martha S. Lubbe, Irma Kotzé, Marike Cockeran

**Affiliations:** 1 Medicine Usage in South Africa (MUSA), Faculty of Health Sciences, North-West University, Potchefstroom, South Africa; 2 Department of Statistics, School of Computer, Statistical and Mathematical Sciences, North-West University, Potchefstroom, South Africa

**Keywords:** incidence, prevalence, HIV or AIDS, medical schemes, South Africa

## Abstract

**Background:**

The South African (SA) private medical schemes environment has over the past two decades respond to the evolving needs of people living with the human immunodeficiency virus (PLWH) or acquired immunodeficiency syndrome (AIDS).

**Objective:**

To determine changes in the incidence and prevalence rate of human immunodeficiency virus (HIV) or AIDS in the SA private medical schemes environment from 2005 to 2015.

**Method:**

In this observational study, a single, pharmaceutical benefit management (PBM) company’s medicine-claims database of members with HIV or AIDS has been retrospectively analysed from January 2005 to December 2015. The cohort includes all patients identified by the HIV or AIDS-related diagnostic ICD-10 codes, B20-B24, who also claimed antiretroviral medication during that period.

**Results:**

From 2005 to 2015, the proportion of HIV or AIDS patients enrolled in the PBM-company increased from 0.63% to 2.10%, and the incidence rate of new cases among the beneficiaries increased 2.3 times. The highest HIV or AIDS prevalence and incidence rates were found in the age group ≥ 40 and < 60 years, followed by the age group ≥ 60 and < 70 years. The highest prevalence rates in 2015 were recorded in Gauteng, namely, 422.4/1000 beneficiaries, followed by Western Cape (149.4/1000), and KwaZulu-Natal (118.4/1000).

**Conclusion:**

There has been an increase in the number of SA-PLWH accessing treatment in the medical scheme environment. The high prevalence of HIV infection among older members should signal concern that HIV-related comorbid conditions are likely to become a growing component of care required by PLWH utilizing the SA private healthcare sector.

## Introduction

Human immunodeficiency virus or acquired immunodeficiency syndrome (HIV or AIDS) is a significant cause of death in Africa, and the fourth-largest cause of death worldwide.^1,2^ Access to antiretroviral treatment (ART) in the South Africa’s public healthcare sector is carefully monitored,^3^ yet much remains unknown regarding the number of HIV-positive individuals on ART outside this sector.^4^ The *South African Medical Schemes Act*, No. 131 of 1998,^4^ ensures that the diagnostic and treatment costs of specified medical conditions are addressed in the so-called, ‘prescribed minimum benefits (PMBs)’ regardless of the benefit-option selected by the member.^4,5^ Prescribed minimum benefits cover conditions that meet the definition of a medical emergency, certain specifically defined medical conditions (*n* = 270) and additional chronic conditions (*n* = 26) as per the Chronic Disease’ List (CDL).^4,5^ Furthermore, medical aid schemes in South Africa have a legal obligation to provide PMBs to their members with HIV or AIDS and to pay in full without recourse to co-payment or deductibles.^4,5^ Since 2005, ART has been included as part of PMBs.^4,5^ These benefits also cover HIV voluntary counselling and testing, pain management in palliative care, preventive therapies, hospitalisation and treatment for HIV or AIDS-related opportunistic conditions.^4,5^

Before 2005, South Africans (SAs) obtained ART privately through disease-management, workplace or community treatment programmes.^5,6^ In 2004, the SA public sector started providing ART to its citizens, and whilst ART has resulted in the global decrease of new HIV infections, it has been accompanied by the increased survival of people living with the HIV (PLWH), that is, an increase in the number of persons on long-term care.^1^ The increase in PLWH is also seen in the SA medical schemes environment.^7,8^ Between 2012 and 2017, the Council for Medical Schemes (CMS) reported that the number of its members on ART increased by 72.4%, that is an average annual growth rate of about 11.51%, to 25.12/1000 beneficiaries in 2017.^7^ In spite of managed HIV or AIDS being ranked fourth after hypertension, hyperlipidaemia and diabetes mellitus on SA-CDL,^7,8^ and being the ‘best managed’ chronic condition in the SA private sector,^9^ the influence of HIV or AIDS remains poorly studied in the private healthcare environment. Understanding the epidemic of HIV or AIDS in this context is important to follow and monitor. Hence, against this background, this observational study sought to determine possible changes in the incidence and prevalence rates of treated SA-PLWH who accessed private medical schemes care from 2005 to 2015.

## Research method and design

### Study design and setting

The study design incorporates a longitudinal and retrospective review of data of an open cohort of PLWH from 01 January 2005 up to 31 December 2015. The data were sourced from a large SA pharmaceutical benefit management (PBM) company with more than 1.8 million beneficiaries in 42 medical schemes and capitation plans. To ensure the quality of its data, the PBM company applies several automated confirmatory validation steps to the data. The cohort includes all its members who claimed ART and whose International Classification of Diseases-10 (ICD-10) diagnostic codes, namely, B20–B24, confirmed the presence of HIV and/or an HIV-related condition. The research database includes only those PBM members who claimed one or more prescriptions during the study period. Table 1 summarises the yearly demographic profile of the study population. The dataset includes the following fields: patient’s demography, namely, date of birth, gender, a unique code for the medical scheme member and beneficiary, prescription number, date of dispensing, trade name of the medication, the National Pharmaceutical Product Index (NAPPI) code of each medicine, the International Statistical Classification of Diseases and Related Health Problems, 10th Revision (ICD-10) code^10^ and name of the province where each item was dispensed.

**TABLE 1:** Demographics of human immunodeficiency virus or acquired immunodeficiency syndrome patients on the pharmaceutical benefit management database from 2005 to 2015.

	2005		2006		2007		2008		2009		2010		2011		2012		2013		2014		2015	
	*n*	%	*n*	%	*n*	%	*n*	%	*n*	%	*n*	%	*n*	%	*n*	%	*n*	%	*n*	%	*n*	%
**Total number of patients on PBM database**																						
Total number of patients (*N*)	1 213 676		1 256 886		910 023		758 497		1 033 039		968 131		864 958		815 789		809 833		838 617		843 972	
Male	537 864	44.32	558 414	44.43	407 955	44.83	343 169	45.24	473 809	45.87	446 744	46.14	402 488	46.53	384 159	46.89	379 756	46.89	392 235	46.77	398 166	47.20
Female	675 812	55.68	698 472	55.57	502 068	55.17	415 328	54.76	559 230	54.13	521 387	53.86	462 470	53.47	431 630	53.11	430 077	53.11	446 382	53.23	445 626	52.80
**Total number of HIV or AIDS patients**																						
Total HIV or AIDS patients (*n*)	7665	0.63	10 177	0.81	10 094	1.11	11 687	1.54	16 035	1.55	19 209	1.98	18 851	2.18	16 075	1.97	16 407	2.10	15 964	1.90	17 302	2.10
Male	3270	42.7	4338	42.6	4149	41.1	5093	46.1	7105	44.30	10 152	52.90	10 300	54.60	8221	51.10	8187	49.90	7537	47.20	8250	47.70
Female	4395	57.3	5839	57.4	5945	58.9	6594	57.9	8930	55.70	9057	47.10	8551	45.40	7854	48.90	8220	50.10	8427	52.80	9062	52.30
**Classification by age groups of HIV or AIDS patients**																						
0 and < 6 years	53	0.69	50	0.49	29	0.29	3	0.03	39	0.24	21	0.11	21	0.11	23	0.14	32	0.20	28	0.18	39	0.22
≥ 6 and < 12 years	349	4.55	448	4.40	436	4.32	424	3.63	473	2.95	446	2.32	380	2.02	340	2.12	347	2.11	329	2.06	326	1.88
≥ 12 and < 18 years	39	0.51	84	0.83	90	0.89	121	1.04	162	1.01	186	0.97	197	1.05	214	1.33	252	1.54	307	1.92	309	1.79
≥ 18 and < 40 years	229	2.99	293	2.87	240	2.38	225	1.93	341	2.13	328	1.71	277	1.47	263	1.64	293	1.79	279	1.75	314	1.81
≥ 40 and < 60 years	5199	67.83	6670	65.64	6395	63.35	7066	60.46	9830	61.30	10 187	53.03	9821	52.10	9070	56.42	9084	55.37	8745	54.79	9742	56.31
≥ 60 and < 70 years	1716	22.39	2555	25.11	2831	28.05	3764	32.21	5085	31.71	5896	30.69	6002	31.84	5973	37.16	6165	37.58	6035	37.80	6307	36.45
≥ 70 years	80	1.04	77	0.76	73	0.72	84	0.72	105	0.65	135	0.70	142	0.75	192	1.94	234	1.43	241	1.51	265	1.53

PBM, pharmaceutical benefit management; HIV or AIDS, human immunodeficiency virus or acquired immunodeficiency syndrome.

### Statistics: Variables and measurements

The number of HIV or AIDS patients on the database was stratified by year, gender, age group and province. The patient’s age was determined at the time of the first dispensing in the index year, namely, 2005. It was thereafter divided into seven age groups: > 0 and < 6 years; ≥ 6 and < 12 years, ≥ 12 and < 18 years; ≥ 18 and < 40 years; ≥ 40 and < 60 years; ≥ 60 and < 70 years; and ≥ 70 years. Patients were also grouped into two categories according to their gender (male and female) and province.

In this study, the *prevalence rate* of treated PLWH was calculated per 1000 medical scheme beneficiaries annually who claimed one or more prescriptions during the specific year^11^:

$$\begin{array}{l} \text{Prevalence rate}=\frac{\begin{array}{l} \text{All new and pre-existing cases}\\ \text{during a given period} \end{array}}{\text{The population during the same period}}(\times 10^{n})\\ n=3 \end{array}$$ [Eqn 1]

The population in Equation 1 includes the total population or the population of specific gender or age group on the database who claimed one or more prescriptions during the specific year.

The *incidence rate* was used to determine the proportion of study participants who had newly registered their HIV or AIDS status with their medical schemes during the study period without taking into account when participants contracted the disease. Each participant was followed from the time he or she was registered with the central database. Participants who cancelled their membership with a specific medical scheme did not contribute to the year’s denominator, whereas new members did.

The HIV or AIDS incidence rate was calculated as the number of new cases per 1000 medical scheme beneficiaries who claimed one or more prescriptions during the specific year. The incidence rate was calculated as follows:^11^

$$\begin{array}{l} \text{Incidennce rate}=\frac{\begin{array}{l} \text{Number of new cases of a disease on}\\ \text{the database in a specified period} \end{array}}{\begin{array}{l} \text{Size of population at start of}\\ \text{the specified period} \end{array}}(\times 10^{n})\\ n=3 \end{array}$$ [Eqn 2]

The population in Equation 2 includes the total population or the population of specific gender or age group on the database who claimed one or more prescriptions during the particular year.

### Data analysis

The Statistical Analysis System^®^ (SAS 9.4^®^) software (SAS Institute Inc., 2002–2012) was used to analyse the data. Variables were expressed using descriptive statistics, which include numbers (*n*) and proportions presented as percentages (%).

### Ethical consideration

This study was approved by the Health Research Ethics Committee of the North-West University (certificate number NWU-00179-14-A1), and the ‘Goodwill Permission’ to perform the study was obtained from the board of directors of the company. All data were anonymised prior to the incorporation in this study.

## Results

The study population and those on the PBM database who claimed one or more prescriptions in a specific year were stratified by gender and age group as shown in Table 1.

In 2005 and 2015, 1 213 676 and 843 972 patients claimed medicines, respectively. In 2005, 7665 (0.63%) of patients on the PBM database were PLWH. This number increased to 17 302 (2.05%) in 2015. In 2005, of the total of 675 812 females, 4395 (0.65%) were PLWH, and of the total of 537 864 males, 3270 (0.61%) were PLWH. In 2015, female patients totalled 445 626, of whom 9092 (2.04%) were PLWH, and of the 398 166 male patients, 8250 (2.07%) were PLWH.

**FIGURE 1:**
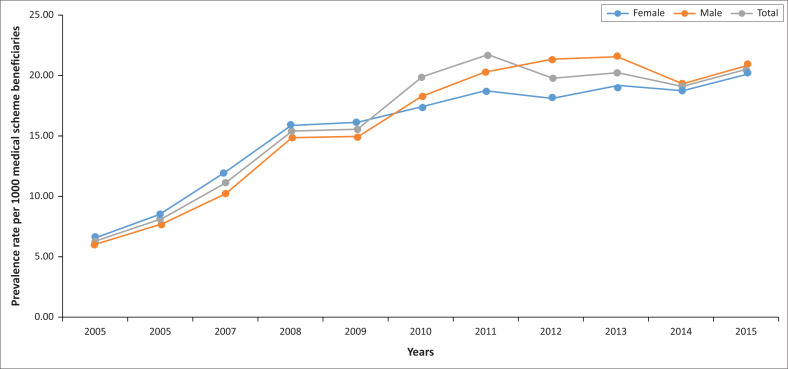
Prevalence rate of human immunodeficiency virus or acquired immunodeficiency syndrome patients per 1000 medical scheme beneficiaries per gender from 2005 to 2015.

The results in Figure 1 show the prevalence rate of HIV or AIDS patients per 1000 medical scheme beneficiaries, which had increased from 6.3/1000 in 2005 to 20.5/1000 in 2015. The prevalence rate of female HIV or AIDS patients was 6.5/1000 in 2005, which increased to 20.4/1000 by the end of 2015. In males, the prevalence rate of HIV or AIDS increased from 6.0/1000 (in 2005) to 21.7/1000 in 2015.

**FIGURE 2:**
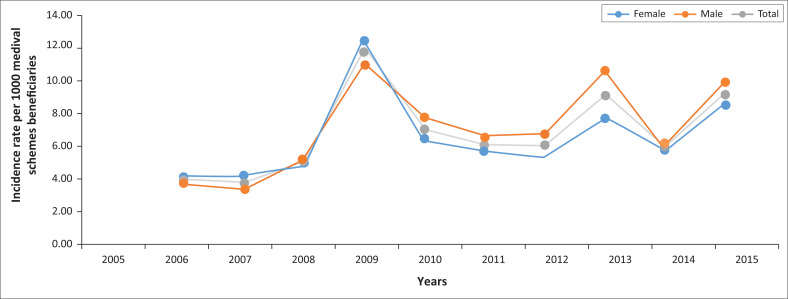
Human immunodeficiency virus or acquired immunodeficiency syndrome incidence rate per 1000 medical scheme beneficiaries per gender in the medical schemes environment of South Africa between 2005 and 2015.

Figure 2 demonstrates change in the incidence rate of new HIV or AIDS cases on the database per 1000 medical scheme beneficiaries from 2005 to 2015. The combined incidence rate increased from 3.9/1000 in 2006 to 9.1/1000 in 2015. The HIV or AIDS incidence in females increased from 4.0/1000 in 2006 to 8.5/1000 in 2015, whilst that of males rose from 3.9 in 2006 to 9.9/1000 in 2015. The intermittent increase (‘spikes’) in the incidence rate of new HIV or AIDS cases on the database of both genders is an artefact that reflects changes during the period in the overall number of members of contributing medical aid schemes.

**FIGURE 3:**
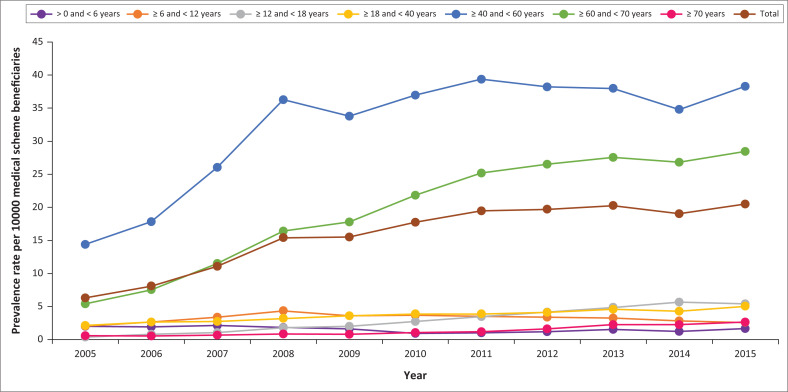
Prevalence rate of human immunodeficiency virus or acquired immunodeficiency syndrome patients per 1000 medical scheme beneficiaries as per age groups from 2005 to 2015.

**FIGURE 4:**
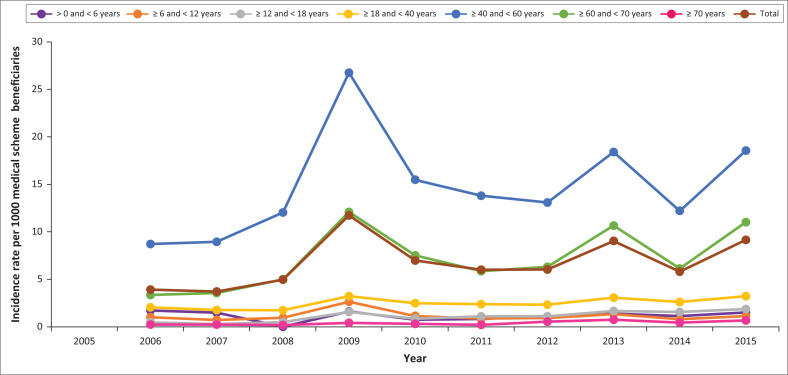
Incidence rate of human immunodeficiency virus or acquired immunodeficiency syndrome per 1000 medical scheme beneficiaries as per age groups from 2005 to 2015.

Figures 3 and 4 illustrate the respective prevalence of HIV or AIDS patients and incidence rates of new HIV or AIDS cases by age on the database per 1000 medical scheme beneficiaries.

Age group ≥ 40 and < 60 years had the highest HIV or AIDS prevalence rates of 14.4 in 2005 and 38.3 in 2015, followed by the age group ≥ 60 and < 70 years. The age group ≥ 0 and < 6 years had the lowest HIV or AIDS prevalence rate followed by the ≥ 6 and < 12 years age group with prevalence rates of 2.1/1000 and 2.6/1000 in 2005 and 2015, respectively. In the age group ≥ 18 and < 40 years, the HIV or AIDS prevalence rate increased by 2.9/1000 beneficiaries between 2005 and 2015. The prevalence rate in elderly patients, ≥ 70 years, was 2.7/1000 in 2015, which is an increase of 2.1 from 2005.

The incidence rate of new HIV or AIDS cases in the < 6 years age group declined from 1.71/1000 in 2006 to 1.51/1000 in 2015. Incidence rates in the age group ≥ 6 and < 12 years remained similar: < 1/1000 beneficiaries in 2006 and 1.1/1000 in 2015, whilst rates in the ≥ 12 and < 18 years and the ≥ 18 and < 40 years age groups increased marginally. The highest increase occurred in the ≥ 40 – ≤ 60-year age group: 8–18/1000 new HIV or AIDS cases. A large increase was also recorded in the ≥ 60 and < 70 years age group: 4–11/1000. The age group ≥ 70 years recorded the lowest HIV or AIDS incidence rate, that is, less than 1/1000 medical scheme beneficiaries in 2006 and 2015. The intermittent ‘spikes’ in the incidence rate are an artefact created by changes in the overall numbers of contributing schemes.

**FIGURE 5:**
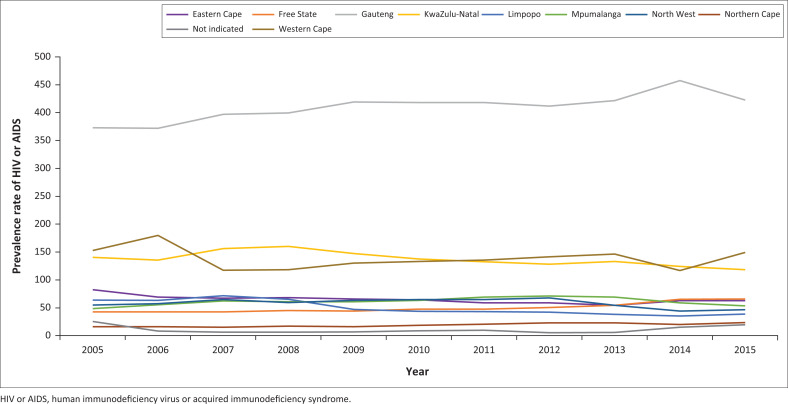
Prevalence rate of human immunodeficiency virus or acquired immunodeficiency syndrome patients per 1000 medical scheme beneficiaries in different provinces of South Africa from 2005 to 2015.

Beneficiary–prevalence data per province are presented in Figure 5. The highest HIV or AIDS prevalence rate was noted in Gauteng at 372.9/1000 in 2005. This increased to 422.4/1000 in 2015. A decline in prevalence rate was noted in KwaZulu-Natal: from 140.4/1000 in 2005 to < 118.4 in 2015. The recorded prevalence amongst beneficiaries increased in both Northern Cape and the Free State, 15.9–23.5/1000 from 42.6–65.5/1000, respectively, in 2015. Prevalence rates have increased in Mpumalanga but decreased modestly in the North West, Limpopo and the Eastern Cape.

## Discussion

This study aimed to identify changes in the SA HIV or AIDS incidence and prevalence rates in a population that was covered by private medical schemes between 2005 and 2015.

Studies determining both incidence and prevalence rates of HIV or AIDS in the medical schemes environment in South Africa are few. Human immunodeficiency virus and ART studies in developed regions, for instance, the United Kingdom and Australia,^12,13^ seldom provide an adequate comparator arm as patients, healthcare systems and individual circumstances are too dissimilar. The results of this study do nonetheless indicate that both incidence and prevalence rates of SA-PLWH who claimed ART through PBM company have increased over the last decade. Specifically, the prevalence rate of HIV or AIDS increased by > 3.3 times and the incidence rate by 2.3 times in this population. These results correlate well with the general data reported by the SA-CMS that confirm the following annual increase in HIV prevalence rates in the managed healthcare sector: 15.36/1000 beneficiaries in 2011, 17.31/1000 in 2015 and 22.08/1000 in 2016.^8,9,14^

Our data confirm the preponderance of females over males – certainly a consequence of the generalised character of HIV epidemic in sub-Saharan Africa. Our results mirror the results of public sector.^15,16^ Yet the results also suggest that the female predominance of earlier incidence studies – a fact that is visible in our data from 2005 to 2009 (Table 1) – has begun to narrow and at times show reverse trend. Is the SA HIV epidemic gradually changing and perhaps entering a new phase? Alternatively, is the nature of employment in the SA private sector currently favouring a greater representation of males? Answers to these questions are currently unclear.

With regard to age, the following points are made. Representation of HIV in the youngest groups, namely, 0–12 years is low. The fact that the prevalence has fallen steadily in the 6–12-year age group as well is testimony to the success of vertical transmission prevention efforts introduced almost 15 years ago. These findings are similar to those reported in the CMS annual report of 2015–2016.^9^ Furthermore, the adults in our cohort are ageing, and those with the highest prevalence rates of HIV are moving out of the child-bearing category, namely, most are aged 40+ years.

A review of the demographics of adolescents (age 10–19 years) and young adults in this study indicates that this group is not represented adequately. This is not surprising for a country (such as South Africa) where one-third of youth aged below 30 years is unemployed and up to 50% of school leavers cannot find work, that is, cannot access private healthcare. This group is nonetheless a high-risk group for HIV transmission, infection and failure of HIV-management.

The highest HIV prevalence occurred amongst middle-aged and older persons, namely, 40–70 years old. Figure 4 clearly indicates that this group has consistently accounted for 80% – 90% of HIV-infected population managed by private medical schemes. Figure 4 also provides graphically the annual new incident infections in this age group. If these data are duplicated in all SA medical schemes, then the private sector must begin to view their 40+-year-old HIV-infected patients as a key population for whom the message of HIV prevention must become a priority.

The 40–70-year age group is at risk of comorbid diseases. These include diabetes, hypertension, renal dysfunction, neurocognitive decline, life-threatening cardiovascular events, fragility fractures and non-AIDS defining cancers. These may occur a decade earlier than occurring in uninfected peers and result from the persistently inflammatory milieu that cannot be corrected currently by ART.^17,18,19^ Our data indicate that the adult HIV-infected group is ageing and is likely to develop one or more comorbid conditions. While this condition cannot be fully reversed, it could be mitigated with, for example, changes in medication, lifestyle and dietary changes, and regular medical assessments to evaluate risk. Long-term consequences of comorbid disease in PLWH means greater exposure to drug–drug interactions, drug toxicities and increased healthcare costs. The 40+-year age group needs to be monitored closely in this regard.

According to the 2017–2018 CMS annual report, maximum number of healthcare service providers, healthcare-related visits and beneficiaries were found in Gauteng, followed by the Western Cape. Mpumalanga, the Northern Cape, Western Cape and Limpopo consistently have lower proportions.^8^ Each of the other provinces made up less than 10% of covered beneficiaries. Disparity in medical scheme coverage according to province is likely to reflect the urban–rural divide, employment status and lack of opportunity of those in rural areas. It is to be noted that membership of a medical scheme is linked with the availability of employment.

## Study’s strengths and limitations

An important limitation of this study is the fact that data were obtained from only one PMB. Generalisations may, therefore, be inappropriate. Furthermore, the data we have been able to obtain are restricted to broad demographic categories and excludes other data with a specific clinical interest, for example, long-term survival, morbidity and so on. The data are also limited by its retrospective nature/capture. Nevertheless, the data provide an important overview of the incidences and prevalence of HIV infection in the SA private sector, namely, a large South African PBM company. The number of PLWH registered with companies that supply PMBs and provide disease management programmes has increased significantly during the last decade, that is, this sector is important for the successful delivery of HIV care to the nation. Indeed, the provision of PMBs – strength of programme – highlights positive benefits that members are able to access.

## Conclusion

Our study indicates an increase in the number of SA-PLWH accessing treatment in private healthcare sector and utilising PMBs. The latter has proven to be successful in managing HIV or AIDS in the private medical schemes’ environment. The growing prevalence of middle-aged and older adults with HIV or AIDS warrants further studies as this group is sexually active and presents an opportunity to re-emphasise HIV-prevention messages. In addition, this group is at risk of comorbid diseases that would affect their risk-profile assessments and their long-term survival.
